# Mitophagy and the mitochondrial unfolded protein response in neurodegeneration and bacterial infection

**DOI:** 10.1186/s12915-015-0129-1

**Published:** 2015-04-03

**Authors:** Mark W Pellegrino, Cole M Haynes

**Affiliations:** Cell Biology Program, Memorial Sloan Kettering Cancer Center, 1275 York Avenue, New York, NY 10065 USA; BCMB Allied Program, Weill Cornell Medical College, 1300 York Avenue, New York, NY 10065 USA

## Abstract

Mitochondria are highly dynamic and structurally complex organelles that provide multiple essential metabolic functions. Mitochondrial dysfunction is associated with neurodegenerative conditions such as Parkinson’s disease, as well as bacterial infection. Here, we explore the roles of mitochondrial autophagy (mitophagy) and the mitochondrial unfolded protein response (UPR^mt^) in the response to mitochondrial dysfunction, focusing in particular on recent evidence on the role of mitochondrial import efficiency in the regulation of these stress pathways and how they may interact to protect the mitochondrial pool while initiating an innate immune response to protect against bacterial pathogens.

Mitochondria are dynamic cellular compartments responsible for numerous essential cellular processes including energy production via oxidative phosphorylation and amino acid and nucleic acid metabolism, as well as the regulation of apoptosis. These double-membrane-bounded organelles are composed of over 1,000 proteins, most of which are encoded in the nuclear genome, with the rest encoded in the mitochondrial genome. As a direct consequence of their function as the site of oxidative phosphorylation, mitochondria are also a primary site of reactive oxygen species (ROS) generation. Combined, these mitochondrial features present considerable challenges both to organelle assembly and to the maintenance of homeostasis. In this review, we focus on the role of mitochondrial import efficiency in the regulation of two mitochondrial stress response pathways and their roles in neurodegeneration and bacterial infection.

Thirteen essential components of the respiratory chain, and ATP synthase, which catalyzes the final step in the generation of ATP, are encoded by the mitochondrial genome (mtDNA). They are translated on mitochondrial ribosomes and directly inserted into the mitochondrial inner membrane [1]. Mitochondrial proteins encoded in the nucleus and translated on cytosolic ribosomes by contrast must be targeted to the mitochondria and subsequently imported (Figure 1A). In most cases the targeting is achieved by an amino-terminal mitochondrial targeting sequence (MTS), although internal sequences also exist [2]. Once at the mitochondrial outer membrane, the protein is directed to the appropriate mitochondrial subcompartment: the outer membrane, intermembrane space, inner membrane or matrix. To enter the matrix, the protein crosses the inner membrane through the TIM (translocase of inner membrane) complex, where the MTS is cleaved and the protein folds and assembles into its functional conformation. Crossing the inner membrane requires both a membrane potential, which is generated by a functional respiratory chain, and molecular chaperones located in the mitochondrial matrix (Figure 1A). Mitochondrial protein import is disrupted in multiple pathologic states, and is emerging as a central regulatory step affecting metabolism and stress responses [3].

**Figure 1:**
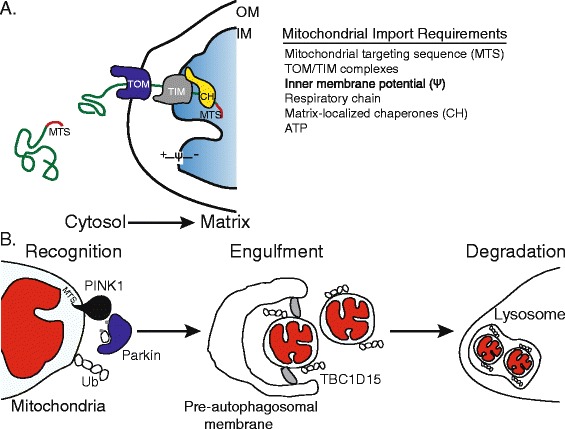
**Mitochondrial protein import and PINK1-mediated mitophagy. (A)** The vast majority of mitochondrial proteins are encoded by nuclear genes, synthesized on cytosolic ribosomes and targeted to mitochondria via mitochondrial targeting sequences (MTS). To reach the mitochondrial matrix, proteins synthesized on cytosolic ribosomes first interact with the translocase of the outer membrane (TOM) and then with the translocase of the inner membrane (TIM). Crossing the inner membrane requires both complexes, a membrane potential (Ψ) across the inner mitochondrial membrane that is generated by the respiratory chain, ATP and molecular chaperones (CH) within the mitochondrial matrix. Once in the matrix, the MTS is typically cleaved, allowing the protein to fold and assemble appropriately. Perturbations to the TOM/TIM complexes, respiratory chain, membrane potential and mitochondrial chaperones results in reduced mitochondrial import efficiency. **(B)** The kinase PINK1 serves to monitor mitochondrial health and initiate mitochondrial degradation when an organelle is severely damaged. Normally, PINK1, localized to mitochondria by its MTS sequence, is efficiently imported into the mitochondrion and subsequently degraded. However, when a mitochondrion is damaged (red), resulting in a depleted inner membrane potential or because of high levels of unfolded proteins in the matrix, PINK1 fails to be imported and accumulates on the mitochondrial outer membrane, allowing recognition of the damaged organelle in a sequence of steps, the first of which is the recruitment of the ubiquitin ligase Parkin to the outer mitochondrial membrane. PINK1 phosphorylates ubiquitin (Ub) and the ubiquitin ligase Parkin, and activated Parkin then ubiquitinates outer mitochondrial membrane proteins, leading to the recruitment of the autophagosome machinery and engulfment of the damaged organelle. Precise engulfment requires the Rab GAP TBC1D15 (shown in grey), which is bound to the mitochondrial outer membrane via interaction with LC3/GABARAP (not shown). The autophagosome then fuses with a lysosome, leading to degradation of the defective mitochondria by the proteases and lipases that reside in lysosomes.

In particular, the upstream regulatory proteins that modulate both mitochondrial autophagy (mitophagy) and the mitochondrial unfolded protein response (UPR^mt^) are regulated by mitochondrial protein import efficiency; thus, because mitochondrial import requires multiple mitochondrial functions, import efficiency can provide a useful readout of individual organelle function as well as the function of the entire organelle pool. We begin by reviewing the role of import regulation in these two stress responses.

## Regulation of mitophagy by mitochondrial protein import efficiency

Mitophagy is a form of autophagy that specifically eliminates damaged mitochondria (Figure 1B). Mitophagy is regulated by the kinase PINK1 and the ubiquitin ligase Parkin, proteins encoded by genes that when mutated lead to early onset Parkinsonism [4-6]. Recognition and ultimately selection of defective mitochondria are initiated by PINK1, which contains an amino-terminal MTS [7]. Normally, PINK1 is imported into mitochondria where the MTS is processed in the matrix and the kinase is ultimately degraded by mitochondrial and cytosolic proteases [8,9]. However, during mitochondrial unfolded protein stress, caused by overexpression of a misfolded mitochondrial protein [10], depletion of a mitochondrial chaperone [11], or when the mitochondrial inner membrane potential is severely depleted, PINK1 fails to cross the inner membrane and integrates into the mitochondrial outer membrane [12,13] (Figure 1B). With its kinase domain exposed to the cytosol [14], PINK1 subsequently recruits Parkin from the cytosol [12,13] and activates its ubiquitin ligase activity by phosphorylating Parkin in its amino-terminal ubiquitin-like domain at serine 65 [15]. PINK1 also phosphorylates free ubiquitin as well as poly-ubiquitin at serine 65, which is also required for Parkin activation [16-18]. Parkin activation requires phosphorylation of its ubiquitin-like domain by PINK1, which allows binding to PINK1-phosphorylated ubiquitin. It is believed that the role of phosphorylated ubiquitin is to interact with Parkin, thus releasing Parkin’s autoinhibitory domain [19] and exposing the ubiquitin ligase active site, allowing the enzyme to ubiquitinate multiple mitochondrial proteins [20]. In addition to phosphorylating free ubiquitin, PINK1 also phosphorylates poly-ubiquitin chains conjugated to mitochondrial proteins by Parkin. This activity generates a feed forward mechanism by recruiting phosphorylated Parkin and amplifying substrate ubiquitination and ubiquitin chain synthesis [21]. The accumulation of poly-ubiquitin chains leads to the disposal of the defective organelle in lysosomes (Figure 1B).

While PINK1 and Parkin are at the core of the mitophagy pathway, a number of additional components, many identified in a recent RNA interference screen [22], are required for their translocation and assembly at the outer mitochondrial membrane. TOMM7, a component of the translocase of the outer membrane (TOM), which mediates translocation of proteins encoded in the nucleus, is required for PINK1 accumulation and stabilization in the outer membrane during mitochondrial dysfunction. Parkin recruitment seems to require both chaperones and metabolic signals. For example, the HSP70 family member HSPA1L and BAG4, a HSP70 nucleotide exchange factor and hexokinases are required for Parkin recruitment, indicating the need for cytosolic chaperone activity [22] and possibly implicating aspects of glucose metabolism in the regulation of mitophagy [23].

Mitochondria, long thought of as discrete organelles, are now known to be a network constantly undergoing fusion and fission, in which segments of the network can be isolated by fission. Mitochondrial fission functions downstream of Parkin recruitment to promote mitophagy. Fission requires the dynamin related protein Drp1 and allows the isolation of severely damaged mitochondrial segments and generation of mitochondria of a size compatible with the autophagosome and lysosome [24-26]. Parkin recruitment promotes fission by ubiquitinating and targeting a mitochondrial fusion component for degradation [27]. The generation of the mitochondria-specific pre-autophagosomal isolation membrane (Figure 1B) is regulated by TBC1D15, a mitochondrial Rab GTPase-activating protein (Rab-GAP) [28] and the mitochondrial outer membrane protein Fis1 [29]. To match autophagosome size to that of its defective mitochondrial cargo, TBC1D15 inhibits or slows Rab7, a GTPase activated by Parkin that promotes autophagosome membrane expansion. TBC1D15 also associates with the mitochondrial outer membrane protein Fis1 and the autophagosomal isolation membrane through interactions with LC3/GABARAP (Figure 1B). Once autophagosome formation is complete, the autophagosome fuses with a lysosome and empties its contents into the lysosome interior, where the damaged mitochondria are degraded.

In addition to the regulation of PINK1 by mitochondrial protein import efficiency, additional layers of mitophagy regulation exist to promote the appropriate activation of Parkin activity. The mitochondrial-localized deubiquitinase USP30 [30] as well as the anti-apoptotic Bcl-2 family proteins were recently shown to antagonize Parkin activity [31,32]. Parkin recruitment to mitochondria leads to the ubiquitination of many mitochondrial proteins. And, ubiquitination of mitochondrial proteins by Parkin is antagonized by USP30, suggesting that the deubiquitinase may limit aberrant ubiquitination, thus ensuring that mitophagy results only in the degradation of defective organelles [31]. Bcl-2 proteins regulate apoptosis, ultimately coordinating permeabilization of the outer mitochondrial membrane and the release of proteins such as cytochrome c from the intermembrane space into the cytosol. In addition to impairing apoptosis, the pro-survival Bcl-2 proteins impair mitophagy by directly binding Parkin and impairing its translocation to defective mitochondria, suggesting the anti-apoptotic proteins promote mitochondrial network integrity by impairing mitophagy and membrane permeabilization in the absence of pro-apoptotic stimuli [32].

## Regulation of the UPR^mt^ by mitochondrial protein import efficiency

The UPR^mt^ is a protective or adaptive transcriptional response that promotes survival during mitochondrial stress or dysfunction. It was first documented in cultured mammalian cells by depleting mtDNA with ethidium bromide [33] or by overexpressing a folding incompetent form of ornithine transcarbamylase and targeting it to the mitochondrial matrix [34]. In response, mitochondrial chaperone and protease genes [35] are transcribed and presumably stabilize the mitochondrial protein-folding environment [36,37].

While induction of a UPR^mt^ in mammals occurs during multiple forms of mitochondrial stress, the means of regulation is unclear. Mitochondria-to-nucleus communication in mammalian cells is thought to require the transcription factor CHOP [34], and more recently, the mitochondrial sirtuin SirT3 was found to regulate UPR^mt^ activity via deacetylation of the transcription factor FOXA3, promoting its accumulation in the nucleus and allowing expression of anti-oxidant genes [38]. The estrogen receptor has also been shown to respond to mitochondrial stress to stabilize the protein folding environment in the mitochondrial intermembrane space through the transcriptional upregulation of mitochondrial protease Omi and increase in cytosolic proteasomal activity [38,39].

**Figure 2:**
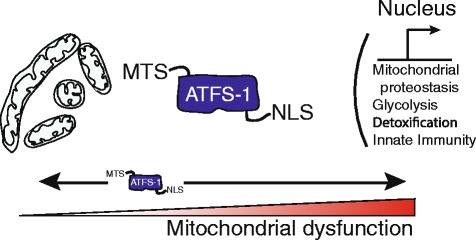
**The ATFS-1-mediated mitochondrial unfolded protein response.** The UPR^mt^ is a protective transcriptional response to the accumulation of unfolded proteins or respiratory chain dysfunction that promotes adaptation and survival during mitochondrial dysfunction. Cells utilize the transcription factor ATFS-1 to monitor mitochondrial function and adjust transcription accordingly. Like PINK1, ATFS-1 is imported into mitochondria and quickly degraded in healthy cells, but accumulates in the cytosol during mitochondrial stress due to respiratory chain dysfunction, unfolded protein accumulation or high levels of reactive oxygen species, when mitochondrial import efficiency is impaired. Because ATFS-1 has a nuclear localization sequence (NLS), as well as its mitochondrial localization sequence (MTS), this causes it to localize to the nucleus, where it induces the transcription of genes involved in mitochondrial protein homeostasis, reactive oxygen species (ROS) and small molecule detoxification, glycolysis, and innate immunity.

Regulation of the UPR^mt^ is much better understood in *Carnorhabditis elegans*, in which it is regulated by the bZip transcription factor ATFS-1, first identified in a genome-wide screen for genes required for the transcriptional induction of mitochondrial chaperones during mitochondrial stress [40]. Like PINK1, ATFS-1 has an amino-terminal MTS and is constitutively imported into healthy mitochondria, where it is processed and degraded by the Lon protease [9,13,41]. However, unlike PINK1, ATFS-1 also has a nuclear localization sequence. Where mitochondrial dysfunction is caused by impairment of mitochondrial chaperones and proteases, mutations to respiratory chain genes [41,42], exposure to respiratory chain inhibitors, including antimycin [43-45], mitochondrial ribosome impairment [46], high levels of ROS, or exposure to ethidium bromide [44,47], mitochondrial import efficiency is slowed [45], causing ATFS-1 to accumulate in the cytosol [41] (Figure 2). Because of its nuclear localization sequence, rather than being degraded in the cytosol [45], ATFS-1 migrates to the nucleus where it induces transcription of over 400 genes that constitute the UPR^mt^. Included in this transcriptional program are mitochondrial proteases and chaperones, mitochondrial fission components, the mitochondrial protein import machinery, anti-oxidant genes and the glycolysis machinery [41].

While many details remain to be elucidated, the transcriptional output of the UPR^mt^ is consistent with a program that promotes survival during mitochondrial dysfunction by adapting metabolism while stabilizing the defective but salvageable organelles to facilitate repair or regeneration.

## Mitophagy and UPR^mt^ interactions

**Figure 3:**
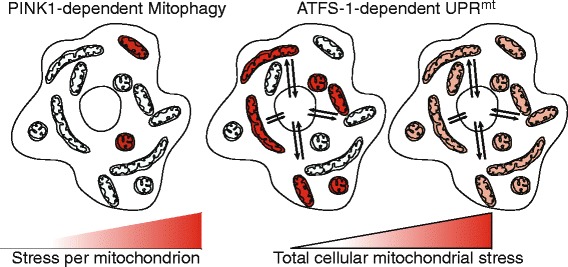
**Integrating mitophagy and the UPR** ^**mt**^ **.** While mitochondria are dynamic, at a given point in time they form a pool of at least semi-independent cellular compartments. Because PINK1 specifically accumulates on individual defective organelles (red), the mitophagy pathway is well equipped to monitor the health of individual organelles. On the other hand, ATFS-1 accumulates in the cytosol in proportion to the total amount of cellular mitochondrial stress. Because mitochondrial protein import is post-translational, ATFS-1 can accumulate in the cytosol during conditions where multiple individual mitochondria are severely defective (red; middle panel), or if the total pool of organelles is modestly stressed (far right panel). ATFS-1 induces transcription of a number of genes whose protein products must be imported into mitochondria to promote their recovery. Because the healthiest organelles are the most import competent, the UPR^mt^ likely promotes the recovery of those organelles that can be salvaged. Concomitantly, by culling the most defective organelles, PINK1-dependent mitophagy enriches the healthy pool of organelles, allowing the resources required for mitochondrial repair to be allocated to the salvageable mitochondrial population.

It is clear that mitochondrial accumulation of PINK1 and activation of ATFS-1 can occur under the same conditions [10,11,41,44,47]; to our knowledge, however, the potential pathway interactions have not been examined simultaneously, although enough data exist to suggest these pathways interact in a complementary fashion. Presumably, mitophagy serves to remove the most severely defective organelles (Figure 3) while the UPR^mt^ promotes stabilization and recovery of those organelles that are salvageable [36]. While mitochondria are known to comprise a dynamic network, at a given point in time they form a pool of at least semi-independent cellular compartments, and it is clear that PINK1 and Parkin specifically accumulate on individual defective organelles: this has been shown in cells bearing a mixture of normal mitochondrial DNA and DNA bearing a deleterious mutation (so-called heteroplasmic cells) [48]. ATFS-1 by contrast serves as a sensor for general levels of mitochondrial stress. Because mitochondrial import is post-translational [49], ATFS-1 accumulates in the cytosol and ultimately the nucleus in proportion to the level of mitochondrial stress throughout the cell. This could reflect a mixture of dysfunctional and healthy mitochondria, or equal levels of stress to all mitochondria (Figure 3). A number of the protein products of genes whose transcription is induced by ATFS-1 must be imported into mitochondria in order to promote organelle recovery. We hypothesize that because the healthiest mitochondria are the most import competent, the UPR^mt^ is likely to promote the recovery of those organelles that can be salvaged. Concomitantly, by culling the most defective organelles, PINK1-dependent mitophagy enriches the pool of healthy (or least defective) organelles, reducing aberrant ROS production and the loss of resources that would be wasted attempting to recover unsalvageable organelles [50]. It will be interesting to see whether there is indeed crosstalk and interaction between the pathways to optimize their complementary effects.

## Parkinson’s disease

Parkinson’s disease is characterized by dopaminergic neuronal death in the substantia nigra region of the brain. The majority of cases of Parkinson’s disease are sporadic and develop late in life, and although there are associations of the disease with exposure to environmental toxins, the underlying causes remain unclear. Roughly 10% of all cases, however, are caused by genetic defects, which lead to early onset Parkinson’s disease. These include mutations in the genes encoding PINK1 and Parkin [4-6]. While mitophagy is well studied in cell culture models, documenting mitochondrial degradation due specifically to PINK1 or Parkin has been difficult *in vivo*, particularly in vertebrates [51]. However, a number of recent studies in mice and rats lacking PINK1 or Parkin have demonstrated physiologic defects consistent with impaired mitophagy. Striatal neurons of Parkin-deficient mice have respiratory chain defects [52] and PINK1-deficient mice have been found to be susceptible to forms of mitochondrial stress such as overexpression of misfolded mitochondrial proteins resulting in neuron loss [53,54]. Significantly, PINK1-deficient rats display nigral degeneration coupled with motor deficits, suggesting this model may be physiologically quite similar to the human mutations [55].

Early studies in *Drosophila* remain the strongest examples of PINK1 or Parkin deficiency causing mitochondrial dysfunction and consequent physiological defects, because in flies these proteins are required for homeostasis even in the absence of stress, and the phenotype is much stronger. Thus, flies lacking either enzyme have severe flight muscle and spermatozoa dysfunction along with defects in mitochondrial morphology, all of which are at least consistent with impaired mitophagy [56-58]. This is also consistent with studies on axons of rat primary neurons showing that PINK1 and Parkin mediate the degradation of defective mitochondria via mitophagy [59]. In axons, mitophagy may be more important than organelle repair, because the axon can extend for a considerable distance from the cell body: lysosome-dependent degradation in these studies occurred in the axon rather than by transport back to the cell body.

In addition to the degradation of whole organelles, PINK1 and Parkin are required for the generation of mitochondria-derived vesicles (MDVs), which transport damaged mitochondrial components to the endomembrane system and ultimately the lysosome for degradation [60-62]. MDVs can contain oxidized material from the mitochondrial outer membrane, but also the inner membrane and matrix. It is thus possible that PINK1 senses localized mitochondrial damage or damaged mitochondrial subcompartments in a manner similar to that by which it regulates mitophagy via mitochondrial import deficiency, but rather than degrade the entire organelle, MDVs are generated to specifically remove the damaged segments. Vps35, a protein that participates in a number of membrane trafficking events, has also been implicated in MDV formation and has recently been linked to Parkinson’s disease [63,64]. Interestingly, a separate study has also implicated PINK1 and Parkin in the selective removal of respiratory chain complexes [65]. It should be noted that in addition to mitochondrial quality control, PINK1 has been found to have roles in mitochondrial dynamics [66-68], mitochondrial transport and positioning in neurons [69,70], and regulating the assembly and activity of complex I of the respiratory chain [71], which may also account for the observed mitochondrial dysfunction in cells and organisms lacking PINK1.

Roles for the UPR^mt^ in Parkinson’s disease are emerging but remain circumstantial, possibly because UPR^mt^ regulation is not well understood in mammals. Compounds or mutations that cause mitochondrial dysfunction and Parkinson’s-like symptoms, such as the respiratory complex I inhibitor rotenone and the superoxide generator paraquat, strongly induce the UPR^mt^ [41,44,72]. And ATFS-1 is required for animal development during these conditions [42], suggesting a protective role during Parkinson’s-associated mitochondrial stress, but considerable work remains to establish a connection.

## Bacterial pathogenesis

A number of bacteria and bacterial pathogens secrete protein or toxin virulence factors that perturb mitochondrial function to promote proliferation and infection [73-79]. For example, species of *Streptomyces* produce the respiratory chain complex III inhibitor antimycin and the ATP synthase inhibitor oligomycin [80], and the human pathogen *Pseudomonas aeruginosa* produces the complex IV inhibitor cyanide as well as a number of iron chelators that also perturb mitochondrial function [81,82]. Conversely, mitochondria have a number of roles in resistance against bacterial infection, including the production of bactericidal ROS [83] and inflammasome activation [84]. Interestingly, recent work from a number of laboratories has indicated protective roles for mitophagy and UPR^mt^ components during bacterial infection.

Work in *C. elegans* has indicated that perturbation of mitochondrial function by exposure to toxins or by RNA interference-mediated knockdown of mitochondrial genes induces an innate immune response [43,85-87]. Exposure to *P. aeruginosa*, which affects mitochondrial function as well as numerous other cellular functions, eventually colonizing the intestinal lumen and killing the worm, induces innate immune responses similar to those induced by mitochondrial dysfunction, suggesting a link between bacterial infection, mitochondrial stress and the UPR^mt^. Indeed, upon exposure to *P. aeruginosa*, mitochondrial chaperone and secreted lysozyme gene transcription is specifically induced in the intestine. The UPR^mt^ is not induced when the worms are exposed to *P. aeruginosa* lacking the global virulence activator gene *gacA*, as well as genes required for cyanide and iron chelator production, indicating that mitochondrial perturbation is caused by pathogen virulence. Importantly, ATFS-1 impairment increases susceptibility to *P. aeruginosa*, which is consistent with a role for ATFS-1 in protective innate immunity. Interestingly, when worms were individually fed over 500 bacterial strains, only 18% caused UPR^mt^ activation [43], including a number of potentially pathogenic bacteria species such as *Pseudomonas* and Enterobacteriaceae, suggesting the possibility that ATFS-1 and the UPR^mt^ is a means to detect and ultimately eliminate those pathogens that target mitochondrial function [87]. Innate immune responses initiated by pathways in place to monitor intracellular dysfunction may be especially important in microbe-rich environments such as the intestine where distinguishing pathogenic from commensal bacteria may be particularly challenging [86,88-90].

In addition to the UPR^mt^, roles for PINK1 and Parkin during pathogen infection have been identified. A study in *C. elegans* demonstrated a requirement for PINK1 and mitophagy in the degradation of those mitochondria damaged by *P. aeruginosa*-produced siderophores [82]. Additionally, multiple gene association studies in patients have shown a link between loss-of-function mutations in the gene encoding Parkin with increased susceptibility to the intracellular pathogenic bacteria *Mycobacterium leprae* and *Salmonella enterica*, which cause leprosy and typhoid fever, respectively [91-93]. More recently, Parkin deficient cells, mice and flies have been found to be susceptible to *Mycobacterium tuberculosis* [94]. *M. tuberculosis* proliferation is increased in macrophages with defective Parkin [94]. And, also in macrophages, Parkin was found to co-localize with the bacteria, where it ubiquitinates phagosomal vesicles, targeting them for lysosomal degradation in a manner strikingly similar to mitophagy. It is currently unclear if PINK1 is required for Parkin’s role in targeting pathogenic bacteria for lysosomal degradation but it seems unlikely as there is no obvious means for PINK1 to interact with the pathogen [95]. It will be interesting to determine how Parkin is localized to intracellular pathogens and if there is another ubiquitin kinase that contributes to activation of Parkin in this context.

## Therapeutic considerations

Several recent discoveries about the regulation of mitophagy and the UPR^mt^ have suggested therapeutic strategies to promote the overall health of the mitochondrial network and limit Parkinson’s disease progression or bacterial infection. While Parkin ubiquitinates defective mitochondria, the mitochondrial deubiquitinase USP30 [30] limits Parkin activity by removing mitochondrial ubiquitin as a form of negative regulation. USP30 inhibition results in increased ubiquitination of dysfunctional mitochondria, resulting in increased mitophagy and protection from mitochondrial dysfunction in cultured mammalian neurons as well as in flies exposed to the mitochondrial toxin paraquat [31]. In an analogous way, mutations that limit mitochondrial import of ATFS-1, thus causing hyper-activation of the UPR^mt^ [96], result in increased innate immune gene and mitochondrial-protective gene expression, yielding increased survival on paraquat and ethidium bromide. Hyper-activation of the UPR^mt^ also increases intestinal pathogen clearance and survival during exposure to *P. aeruginosa* [87].

Several recent reports have demonstrated multiple means by which the defects associated with PINK1 deficiency can be alleviated, suggesting strategies to improve PINK1 function in patients with PINK1 loss-of-function mutations, but also in Parkinson’s patients with an intact mitophagy pathway. PINK1, for example, has been reported to be the rare, and perhaps the only, kinase that preferentially accepts the ATP analog kinetin triphosphate (KTP) with higher catalytic efficiency than its normal substrate ATP *in vitro* [97]. Impressively, treatment of cells with the clinically available KTP precursor kinetin resulted in increased intracellular KTP, which alleviated many of the defects found in PINK1-deficient cells, including Parkin recruitment, mitophagy, and mitochondrial motility.

The main focus of this article has been the mechanisms of mitochondrial quality control; but as mentioned earlier, PINK1 is also important for other aspects of mitochondrial function. Two of these in particular have been the target of alternative strategies for correcting PINK1-deficiency that are aimed not at promoting the destruction of defective mitochondria but at improving or altering mitochondrial function. In one case, the target has been respiratory competency, to which PINK1 is believed to contribute. As in bacteria, vitamin K2 promotes electron transfer in the metazoan respiratory chain, and by taking advantage of the strong *Drosophila* PINK1-deficient phenotype, it has been possible to show that overexpression of the gene *Heix* or *UBIAD1*, which is involved in vitamin K2 synthesis, can correct many of the defects consequent on PINK1 deficiency [98]. In a second case, the target is nucleotide metabolism, whose disruption is also implicated in the consequences of PINK1 deficiency. In Parkinson’s disease patients, transcripts of proteins involved in nucleotide metabolism are increased in neurons. This has recently been shown also to occur in the neurons of PINK1-deficient flies [99], possibly driven by a stress response similar to a UPR^mt^ [100]. Overexpression of these genes as well as pharmacological enhancement of the nucleotide salvage pathways by folate treatment (or supplement with all four nucleotides) rescued the mitochondrial impairment in PINK1-deficient flies, suggesting therapeutic potential [99].

Considerable insight has been gained into how cells cope with mitochondrial dysfunction, but as we continue to learn more about these and other pathways, there is little doubt that more therapeutic avenues will open. In the future, it will perhaps be interesting to combine strategies to simultaneously activate both mitophagy and the UPR^mt^ [99] to determine if positive effects of each can be enhanced.

## Note

This article is part of the series on mitochondria edited by Martin Brand, Navdeep Chandel, Andrew Murray, Jodi Nunnari and Peter Walter. Other articles in this series can be found at http://www.biomedcentral.com/series/Mitochondria
